# Monochromatic green light stimulation during incubation shortened the hatching time via pineal function in White Leghorn eggs

**DOI:** 10.1186/s40104-020-00539-x

**Published:** 2021-02-02

**Authors:** Panlin Wang, Yanyan Sun, Yunlei Li, Jing Fan, Yunhe Zong, Adamu Mani Isa, Lei Shi, Yuanmei Wang, Aixin Ni, Pingzhuang Ge, Linlin Jiang, Shixiong Bian, Hui Ma, Zhengdong Yuan, Xiaolin Liu, Jilan Chen

**Affiliations:** 1 Institute of Animal Sciences, Chinese Academy of Agricultural Sciences, Key Laboratory of Animal (Poultry) Genetics Breeding and Reproduction, Ministry of Agriculture and Rural Affairs, Beijing, 100193 China; 2 College of Animal Science and Technology, Northwest A&F University, Yangling, 712100 China; 3 Beijing DQY Agricultural Science and Technology Co., Ltd., Beijing, 100094 China

**Keywords:** Circadian rhythm, Hatching time, Insulin-like growth factor 1, Melatonin, Monochromatic green light, Pineal gland

## Abstract

**Background:**

Effect of monochromatic green light illumination on embryo development has been reported in chickens. The avian pineal gland is an important photo-endocrine organ formed by a mediodorsal protrusion during embryonic development. However, the involvement of pineal gland in the light transduction process remains to be elucidated. In the present study, we investigated the influence of monochromatic green light on hatching time and explored the possible mechanism via pineal function.

**Results:**

A total of 600 eggs of White Leghorn (Shaver strain) were incubated under photoperiods of either 12 h of light and 12 h of darkness using monochromatic green light (12L:12D group) or 24 h of darkness (0L:24D group) for 18 d. Compared to 0L:24D group, the green light stimulation shortened the hatching time without extending the hatch window or impairing hatchability. The liver of embryos incubated in the 12L:12D light condition was heavier than those of the 0L:24D group on d 21 post incubation which may be linked to the observed increase in the serum concentration of insulin-like growth factor 1 (IGF-1); primary secretion of the liver. Histological structure analysis of pineal gland demonstrated that the light stimulation increased follicle area, wall thickness and lumen area on d 10 and d 12 post incubation. Rhythmic function analysis demonstrated that three clock related genes (brain and muscle ARNT-like-1, *BMAL1*; circadian locomotor output cycles kaput, *CLOCK*; and cryptochrome-1, *CRY1*) and a melatonin rate-limiting enzyme related gene (arylalkylamine N-acetyltransferase, *AANAT*) were rhythmically expressed in the pineal gland of the 12L:12D group, but not in the 0L:24D group. Simultaneously, the light stimulation also increased the concentration of melatonin (MT), which was linked to hepatocyte proliferation and IGF-1 secretion in previous studies.

**Conclusions:**

The 12L:12D monochromatic green light stimulation during incubation shortened hatching time without impairing hatching performance. Pineal gland’s early histological development and maturation of its rhythmic function were accelerated by the light stimulation. It may be the key organ in the photo-endocrine axis that regulates embryo development, and the potential mechanism could be through enhanced secretion of MT in the 12L:12D group which promotes the secretion of IGF-1.

**Supplementary Information:**

The online version contains supplementary material available at 10.1186/s40104-020-00539-x.

## Background

Avian species have a broader visible light spectrum [1, 2] and special extra-retinal photoreceptor in pineal gland [2, 3]. They are more sensitive to environmental lighting than mammals. In commercial poultry hatcheries, fertile eggs are incubated in complete darkness, which is different from the settings in natural incubation. There are evidences that light can increase both body weight and breast muscle weight at the embryonic and post-hatching periods [4, 5]. Furthermore, monochromatic green light was shown to promote the growth of spleen [6] and skeletal muscle [7] in broilers. Additionally, some studies have reported that light stimulation using light emitting diode (LED) during embryogenesis shortened the hatching time [8, 9]. The stimulating effect of monochromatic green light on skeletal muscle was linked to enhanced circulation levels of insulin-like growth factor 1 (IGF-1) in blood [7, 10, 11]. Classically, IGF-1 is a ubiquitous peptide secreted mainly in the liver and has been tied to growth hormones, and considered as a surrogate marker for overall growth hormone status [12]. IGF-1 may therefore play an important role in the light-mediated advanced development of chick embryo.

The avian pineal gland is a derivative of the posterior roof of the diencephalon, and a photo-endocrine organ which plays key role in transducing light information to whole body physiology. It was reported that environmental factors could alter the morphology of the pineal gland. For example, great variations in pinealocyte architecture were observed between nocturnal and diurnal birds [13]. Largest pineal gland was recorded in newborn South Pole seals [14], and it contributes to survival in the harsh environment [15]. Pineal gland reflects a high degree of environmental adaptive plasticity not only in histology but also in rhythmic function [13]. Clock-related genes, including brain and muscle ARNT-like-1 (*BMAL1***)**, circadian locomotor output cycles kaput (*CLOCK***)**, and cryptochrome-1 (*CRY1*), and the melatonin (MT) rate-limiting enzyme related gene of arylalkylamine N-acetyltransferase (*AANAT*) played crucial roles in establishing circadian rhythm within pineal cells and showed rhythmic oscillations under light-dark cycles in adult chickens [16]. Thus far, our knowledge about the histology and rhythmic function of pineal gland during embryogenesis in chickens is still insufficient, especially the comparison effects under various light treatments. Above all, whether the pineal gland is involved in light transduction process during embryogenesis and its potential link with IGF-1 is still unknown.

The objective of this study was to investigate the effect of 12L:12D monochromatic green light stimulation during embryogenesis on hatching time and hatching performance in White Leghorn (Shaver strain), and explore the possible mechanism with the involvement of pineal gland.

## Materials and methods

### Experimental design

A total of four incubators (NK-hatching; Beili Incubation Equipment Co. Ltd., Sichuan, China) used in the experiment were blacked out with shade cloth. Two were outfitted with monochromatic green LED strips (ND-BIS-2D-D10W, Nodark Biolight Technology Co., Ltd., Wuxi, China) (λ = 520–525 nm) while the other two were left with no light source. Fertile eggs were obtained from White Leghorn layers (Shaver strain) of 50 weeks of age. The pure line chickens were sourced from the University of Guelph, Canada and housed in the experimental farm of the Institute of Animal Sciences, Chinese Academy of Agricultural Sciences, Beijing, China. A total of 600 eggs of normal size with an average weight of 62.5 ± 3.0 g were selected and stored for no longer than 7 d at 15 °C and 70–75% relative humidity. Eggs were randomly allocated to the two groups, with two incubators (replicates) per group, and 150 eggs per incubator. Eggs of one group were incubated in monochromatic green light-fitted incubators with a light:dark schedule of 12L:12D and light intensity of 200 lx during the first 18 d of incubation, namely 12L:12D group. Eggs in the control group were incubated in complete darkness and named 0L:24D group. The temperature and humidity were calibrated by a standard thermometer and hygrometer for the four incubators before incubation and monitored every 2 h during the whole incubation period. The incubation was maintained at a temperature of 37.8 ± 0.10 °C, and a relative humidity around 60% until d 18. The incubator temperature changes on d 7 post incubation were showed as an example (Additional file 2: Fig. S1.). From d 19, eggs were transferred to hatching baskets and a temperature of 37.2 ± 0.10 °C and relative humidity of 70% was set for the hatcher. At the end of embryonic d 17, 18, 19, 20, and 21, three embryos or chicks were randomly selected and weighted from each replicate (incubator). The hearts, livers, and pectoral musscles were isolated and weighed to track the embryo and organ development. The weight was calculated using the average weight of each replicate for the statistical analysis.

### Hatching time and hatching performance

From d 19 post incubation, all eggs were transferred to the same hatcher (Haijiang Hatching Equipment Co., Ltd., Beijing, China) in constant darkness until hatching. The number of hatched chicks was counted every 2 h from 468 h to 512 h post incubation to monitor the hatching time for each egg and assigned to the nearest time of hatch. Average hatching time, time to 90% hatch, peak hatching period, and hatch window were recorded for each replicate. Average hatching time was calculated as the mean duration from egg setting to the emergence of chicks for all hatched chicks in a given batch. Time to 90% hatch was the hatching time that 90% of the chicks hatched. Peak hatching period was defined as the duration that 30% to 70% chicks hatched [17]. Hatch window was defined as the time interval from the emergence of the first chick to the last chick [17, 18]. Hatchability was calculated as a percentage of fertile egg number. Every hatched chick was assessed for chick quality within 4–6 h after hatching according to a standardized method of scoring in a scale of 100 [19].

### Histology of pineal gland

To explore the possible mechanism that monochromatic green light affects hatching time, another hatching trial was performed using the same incubation conditions and light treatments. Pineal glands were extracted from six birds each, in the 12L:12D and 0L:24D groups on d 10, 12, 14, 16, and 18 post incubation. The tissues were immediately fixed in 10% neutral buffered formalin. On the 7th day after fixation, all samples were dehydrated in a graded series of alcohols, embedded in paraffin, and dissected into 4 μm thick. These sections were stained with Harris haematoxylin and eosin for histomorphology observation using a Zeiss Axioskop microscope (Carl Zeiss, Thornwood, NY, USA) equipped with a QICam digital camera and NIS Elements software (Nikon Instruments, Melville, NY, USA). The pineal follicle related indicators, including wall thickness (WT), follicle (FL) area, and lumen (LM) area were measured using Digimizer Image Analysis software (Ostend, Belgium).

### Expression of clock-related and *AANAT* genes in the pineal gland

The expression of clock-related genes (*BMAL1*, *CRY1* and *CLOCK*) and the melatonin rate-limiting enzyme related gene (*AANAT*) in the pineal gland was measured using real-time quantitative RT-PCR. Pineal glands were collected every 4 h over a 28-h period from d 17 to d 18 after incubation (zeitgeber time (ZT) 2, 6, 10, 14, 18, 22, and 26, where ZT0 is the time when the light is turned on, ZT12 is the time when the light is turned off). Pineal glands removed during dark period were obtained from birds euthanized and decapitated under dim red light. Six pineal glands were collected at each time point for each group, and the sampling time of 0L:24D group was synchronized with those of 12L:12D group. Two pineal glands were pooled and the total RNA of three pooled samples for each group were extracted with TRIzol reagents following the manufacturer’s protocol (Tiangen Biotech, Beijing, China). cDNA was generated by reverse transcription using 1 μg total RNA in a total of 20 μL reaction volume following the instruction of the manufacturer (TaKaRa, Shiga, Japan). Primer 6.0 was used to design primer sequences for the target genes (Additional file 1: Table S1). Real-time PCR amplification was performed using 1.5 μL cDNA solution per 10 μL reaction volume with SYBR® FAST qPCR Kit Master Mix (Kapa Biosystems, Wilmington, USA). The relative expression of target genes was calculated using glyceraldehyde-3-phosphate dehydrogenase (*GAPDH*) as an endogenous control by the 2^−ΔΔC(T)^ method [20]. Samples were run in triplicate.

### Serum concentrations of melatonin and IGF-1

Serum concentrations of MT and IGF-1 on d 19 post incubation were analyzed. Circadian time (CT) is a standard marker of time that is based upon the oscillation or rhythm in constant darkness. CT0 and CT12 correspond to the beginning time of the subjective day and night respectively and CT26/2 is the time point of CT2 on the second day. The blood samples of twelve embryos were collected from allantois vein after the birds were euthanized by cervical dislocation every 4 h from CT2 to CT26/2. The sampling time of embryos was synchronized in the treatment and control groups. Serum was decanted after 2 h of collection at room temperature. Serum of four embryos were pooled and three pools from each group were subjected to MT and IGF-1 hormonal assay using a commercial ELISA kits (Horabio Biotechnology Co. Ltd., Shanghai, China).

### Statistical analysis

The data of related characteristics of hatching time, hatching performance, pineal follicle histological features, serum hormones, and embryo growth were analyzed using *t*-test using SAS software (SAS 9.2, SAS Institute Inc., Cary, NC, USA). All data in percentage were arcsine-transformed before analysis. Threshold for significance of difference was set at *P* < 0.05. The rhythm of genes was analysed by GraphPad Prism software using the cosinor formula *y* = *c* + *a* × cos [2 (*t*−*ø*)/24], where *c*, *a* and *ø* denote the mesor, amplitude and phase of the cosine wave, *t* is time in hours, and R^2^ is the fitting degree. Statistically significant differences in gene rhythms were indicated by *P* < 0.05 and calculated using the website https://www.danielsoper.com/statcalc/calculator.aspx?id=15 [21].

## Results

### Hatching time and hatching performance

**Fig. 1:**
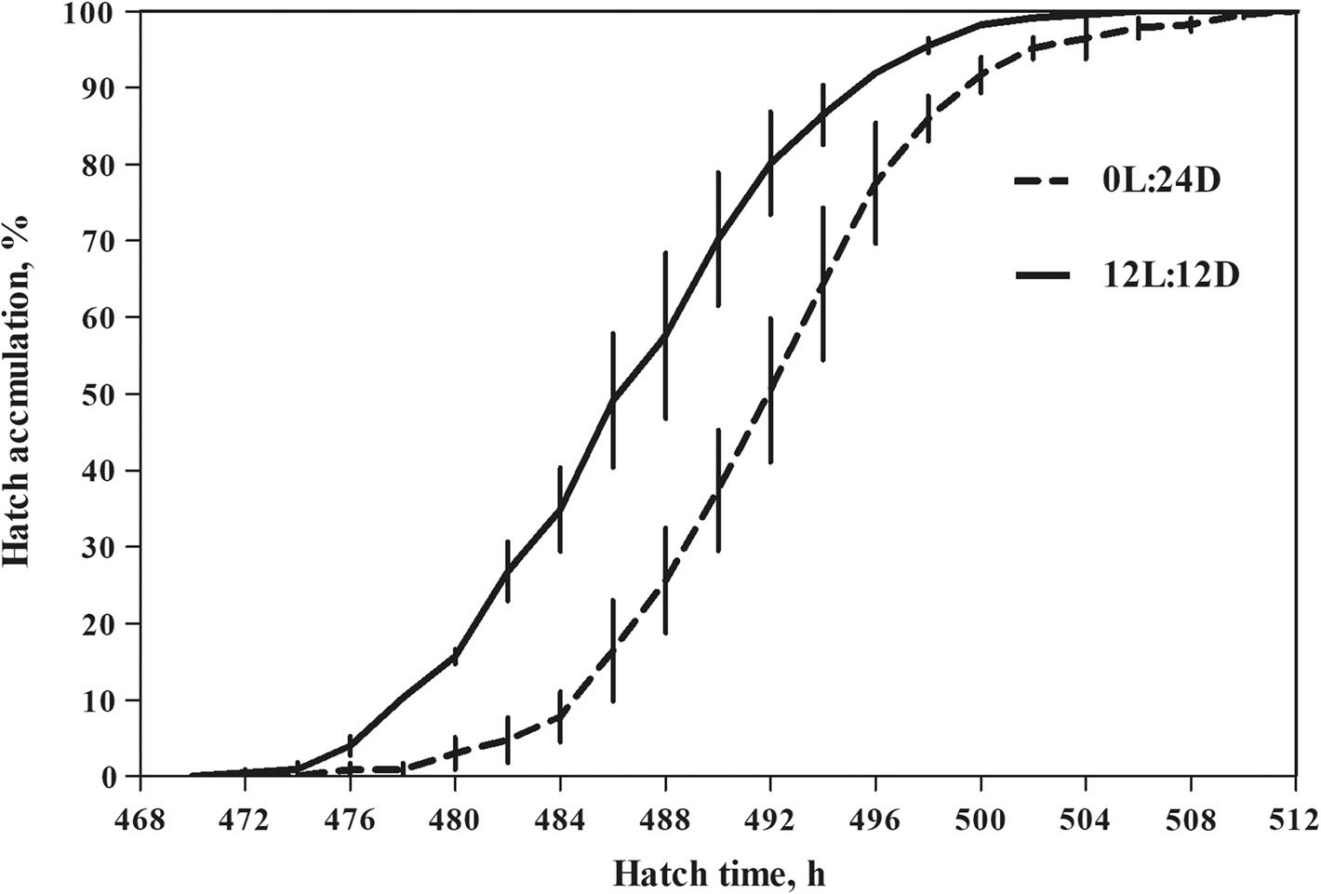
Hatch accumulation in 0L:24D and 12L:12D groups. Data are presented as mean ± SEM (*n* = 2 per group)

The distributions of hatching time are shown in Fig. 1. The 12L:12D group was found to give the first hatching. As shown in Table 1, average hatching time and time to 90% hatch were shorter in 12L:12D group as compared with 0L:24D group (*P* = 0.04; *P* = 0.04 respectively). However, hatch window and peak hatching period were not affected by the light stimulation (*P* = 0.29; *P* = 0.70, respectively). Similarly, hatchability and chick quality score were not affected by light treatment (*P* = 0.72; *P* = 0.61, respectively).

**Table 1:** Effects of light treatments on hatching time and hatching performance

Item	Light treatment		SEM	*P*-value
	0L:24D	12L:12D		
Average hatching time^1^, h	492.92^a^	487.58^b^	1.60	0.04
Time to 90% hatch^2^, h	500.00^a^	494.50^b^	1.65	0.04
Peak hatching period^3^, h	6.50	6.75	0.24	0.70
Hatch window^4^, h	33.00	31.00	0.82	0.29
Hatchability^5^, %	84.81	85.43	0.65	0.72
Chick quality score^6^	97.01	98.02	0.75	0.61

Data are the mean of two replicates

^a, b^ Values within a row with different superscripts differ significantly at *P* < 0.05

^1^ Average hatching time was calculated as the mean duration from egg setting to the emergence of chicks for all hatched chicks in a given batch

^2^ Time to 90% hatch was the hatching time that 90% of the chicks hatched

^3^ Peak hatching period was defined as the duration that 30% to 70% chicks hatched

^4^ Hatch window was defined as the time interval from the emergence of the first chick to the last chick

^5^ Hatchability was calculated as a percentage of fertile egg number

^6^ Chicks were scored for their activity, appearance (plumage, eyes, and legs), and navel area (cicatrisation, retracted yolk, remaining membrane, and yolk) within a total score of 100 [19]

### Embryo and organs development

**Fig. 2:**
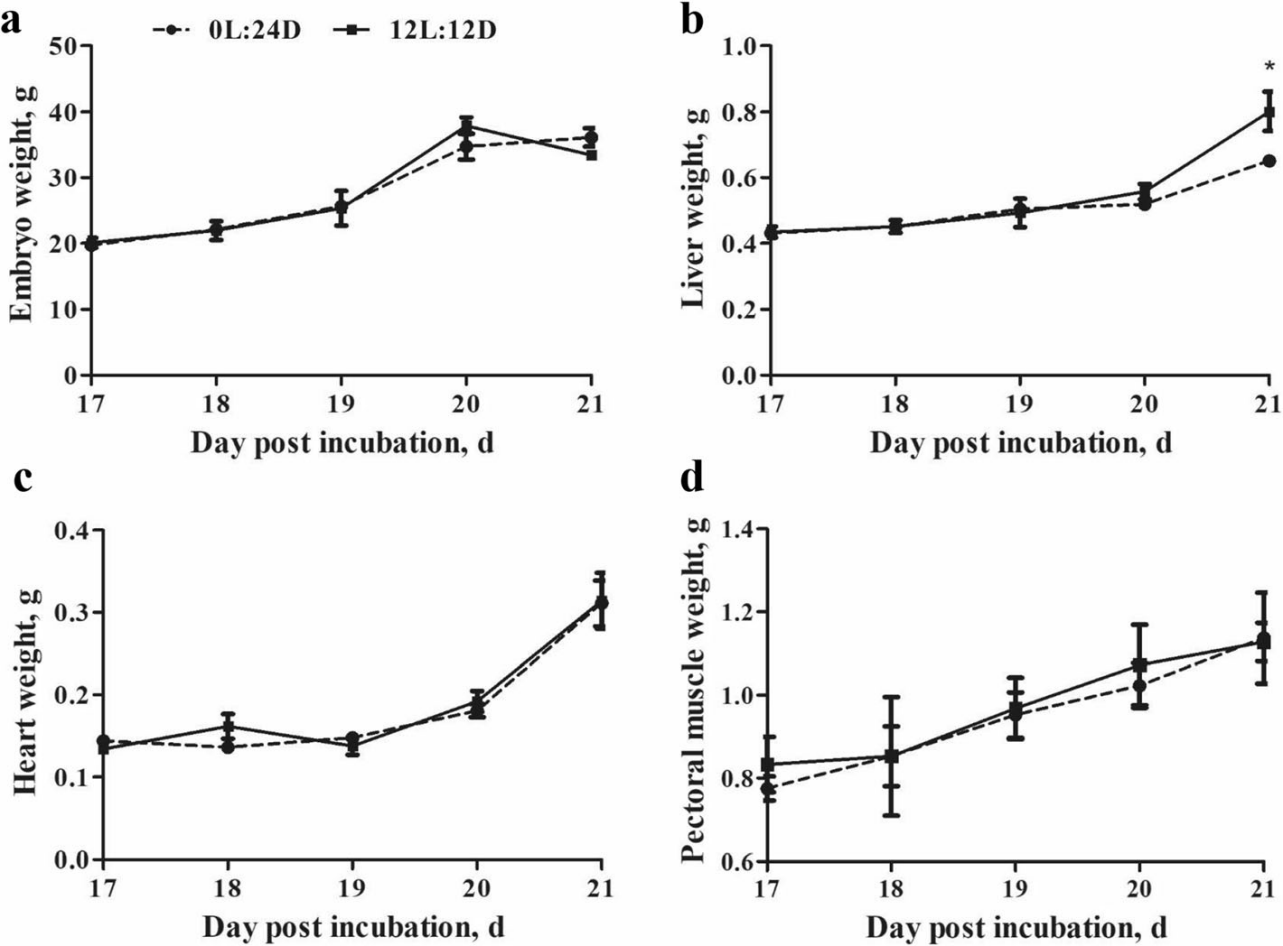
Effect of light treatment on the weight of embryo (**a**), liver (**b**), heart (**c**), and pectoral muscle (**d**) from d 17 to 21 post incubation. Asterisk indicates significant difference between groups at a given incubation stage (* *P* < 0.05). Data are presented as mean ± SEM (*n* = 2 per group)

The weight of embryo, liver, heart and pectoral muscle at different incubation stages are shown in Fig. 2. The results indicate that the light treatment had no effect on the weight of embryo (Fig. 2a), heart (Fig. 2c) or pectoral muscle (Fig. 2d) from d 17 to 21 post incubartion (*P* > 0.05). However, increase in liver weight was observed on d 21 post incubation of the 12L:12D group (*P* = 0.01) (Fig. 2b). Liver, heart, and pectoral muscle weight increased as embryo aged, while embryo weight dropped on d 21.

### Histology of pineal gland

**Fig. 3:**
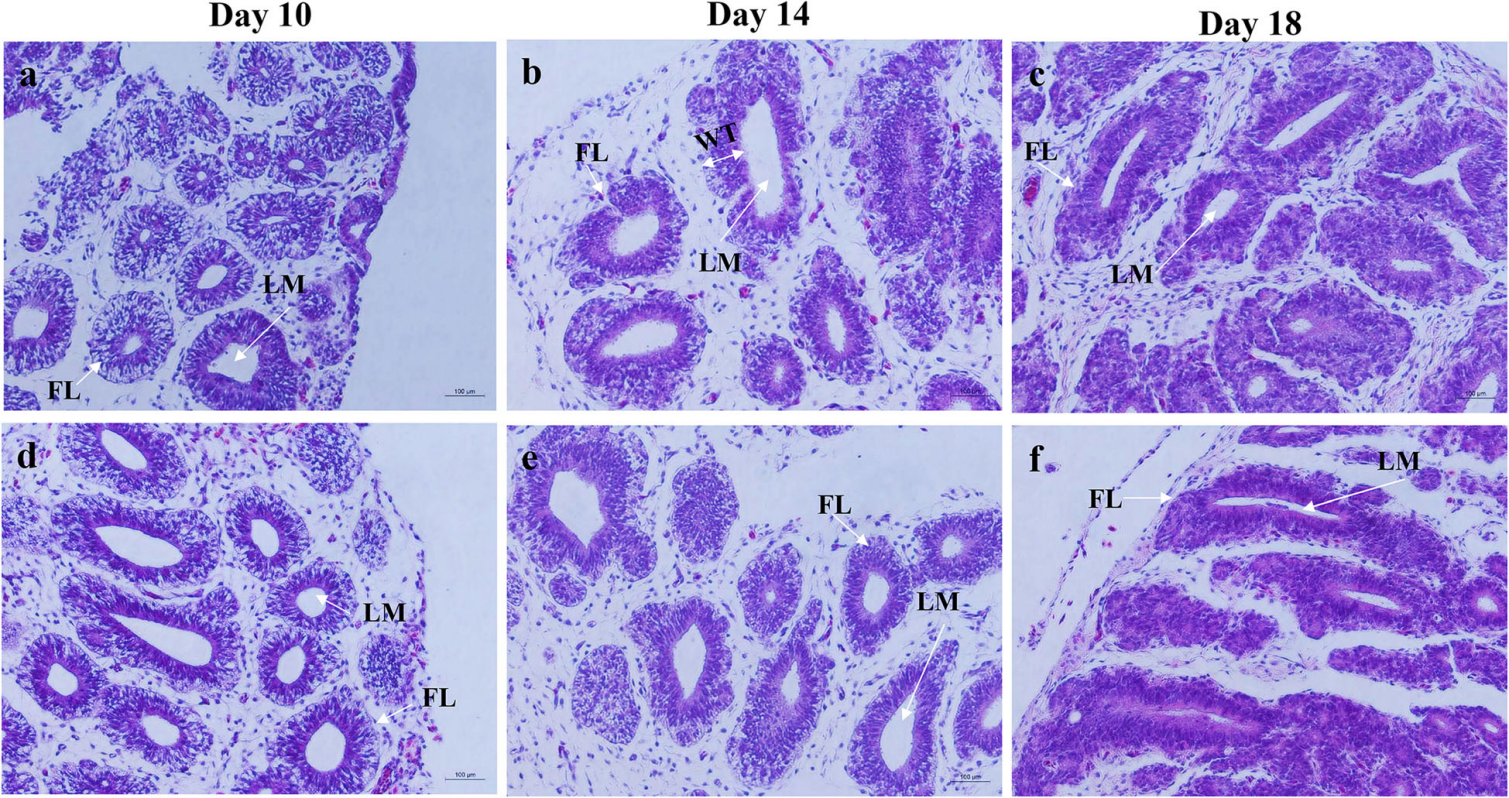
Histological structure of embryonic pineal gland on d 10 (**a**), 14 (**b**) and 18 (**c**) post incubation in the 0L:24D group **d**, **e** and **f** represented the counterpart in the 12L:12D group. FL, follicle; LM, lumen; WT, wall thickness.

**Fig. 4:**
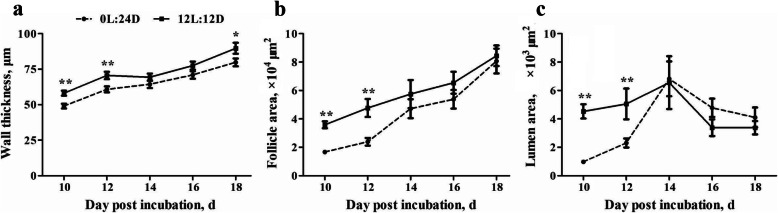
Effect of monochromatic green light stimulation on the histological structure of pineal gland follicle related indicators including wall thickness (**a**), follicle area (**b**), and lumen area (**c**) from d 10 to 18 post incubation. The data are presented as mean ± SEM (*n* = 6 per group). * *P* < 0.05; ** *P* < 0.01

To investigate the effects of monochromatic green light on the structure of pineal gland during embryogenesis, histological observations were performed every 2 days from d 10 to 18 post incubation. The morphological structure on d 10, 14, and 18 post incubation are showed in Fig. 3. Follicle area and wall thickness increased with advancing embryogenesis, while lumen area increased from d 10 to 14 post incubation, but decreased thereafter in both groups (Fig. 4). The 12L:12D monochromatic green light stimulation increased follicle area, wall thickness, and lumen area on d 10 and 12 post incubation (*P* < 0.01). Moreover, d 18 post incubation also witnessed thicker walls in the 12L:12D group (*P* = 0.04).

### Expression of clock-related and *AANAT* genes in the pineal gland

**Fig. 5:**
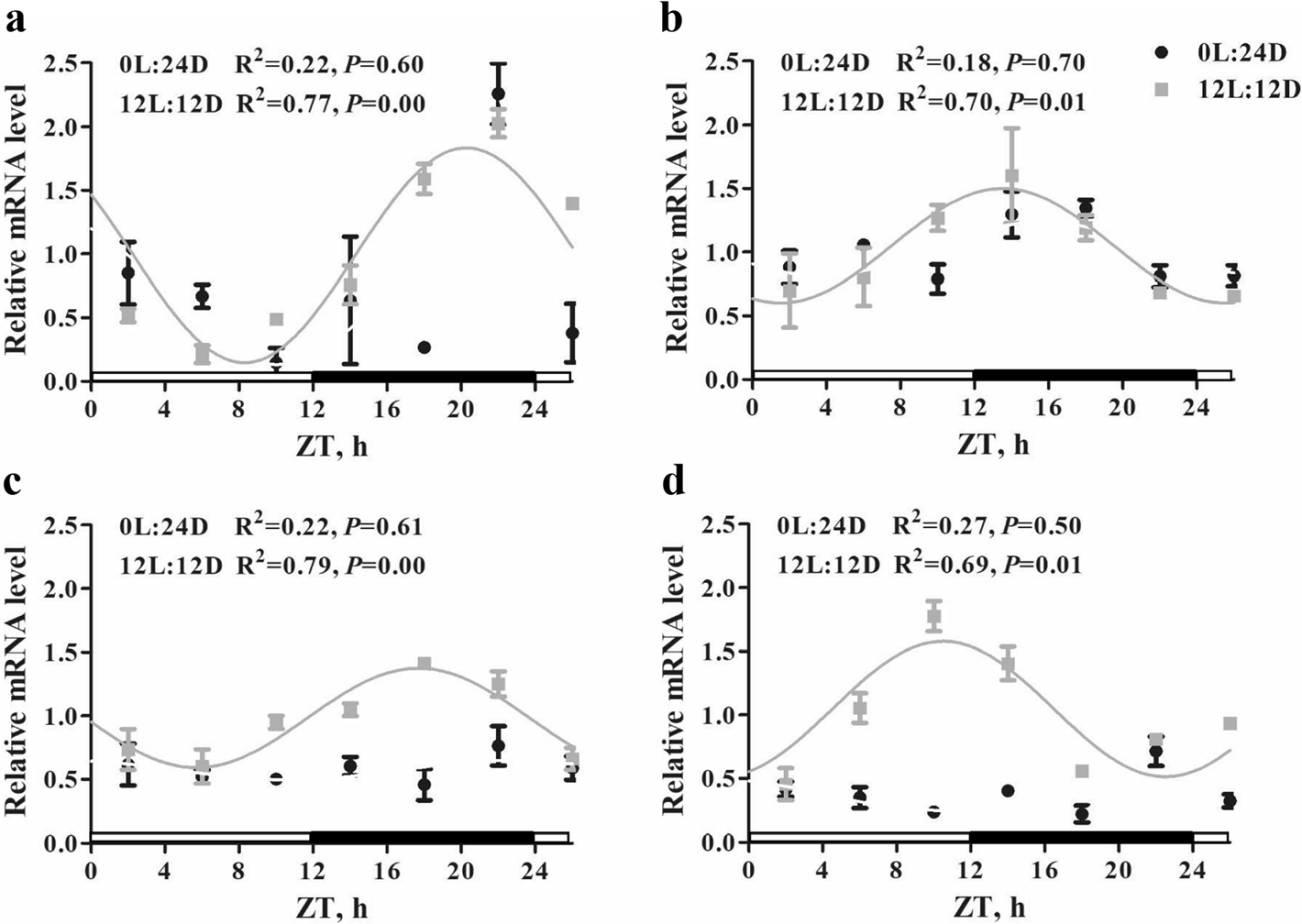
Temporal changes of mRNA levels of target genes in the pineal gland. **a**, arylalkylamine N-acetyltransferase (*AANAT*); **b**, brain and muscle ARNT-like-1 (*BMAL1*); **c**, circadian locomotor output cycles kaput (*CLOCK*); **d**, cryptochrome-1 (CRY1). The horizontal white bar of the X-axis represents light condition, and the black bar represents dark condition in 12L:12D group, while 0L:24D group is in constant darkness throughout the whole day and it is not shown in the figure. The data are presented as mean ± SEM (*n* = 3 per group). The curve shows the best fit to the points by cosinor analysis. The solid line represents rhythmical gene expression, and no solid line represents arrhythmical gene expression. R^2^ values represent the fitting degree. *P* values indicate the significance of cosinor regression analysis. Significance of difference was set at *P* < 0.05. ZT, zeitgeber time

Transcripts of the melatonin rate-limiting enzyme related gene (*AANAT*) and three clock-related genes (*BMAL1, CLOCK,* and *CRY1*) displayed day–night variations from d 17 to 18 post incubation in both groups (Fig. 5). The cosinor analysis showed that *AANAT*, *BMAL1, CLOCK,* and *CRY1* exhibited significant circadian rhythm in the 12L:12D group (*P* = 0.00; *P* = 0.01; *P* = 0.00; *P* = 0.01, respectively), but not in the 0L:24D group (*P* > 0.05).

### MT and IGF-1 hormones

**Fig. 6:**
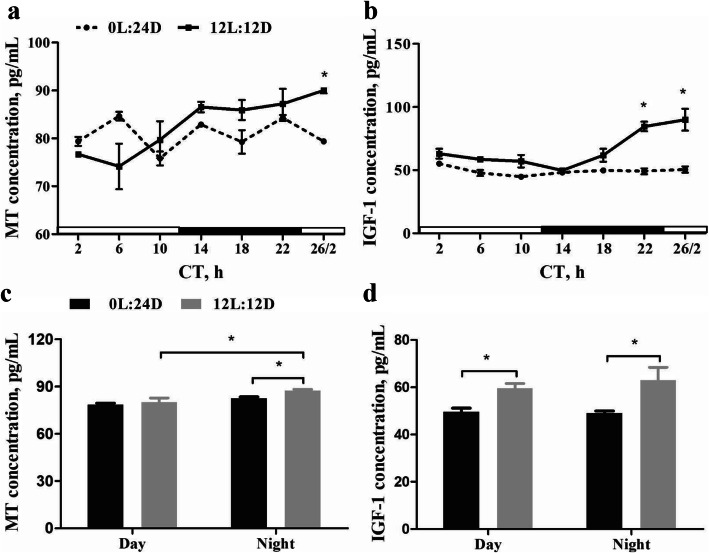
The serum concentrations of MT and IGF-1. Line charts representing temporal changes of MT (**a**) and IGF-1 (**b**) levels. Histogram representing the concentrations of MT (**c**) and IGF-1 (**d**) at the subjective day and night in groups of 0L:24D and 12L:12D. The horizontal white bar represents the subjective day, and the black bar represents the subjective night for the 12L:12D group. The results are presented as mean ± SEM (*n* = 3 per group). Each column represents as mean ± SEM. * *P* < 0.05. CT, circadian time. Day, subjective day; Night, subjective day

Compared with the 0L:24D group, a sharp change of MT levels was observed in the 12L:12D group over the 28 h period of the hormonal monitoring (Fig. 6a). MT levels at subjective night were higher than those at subjective day in the 12L:12D group (*P* = 0.00), but there was no significant diurnal difference in the 0L:24D group (*P* = 0.24) (Fig. 6c). At subjective night, MT levels were higher in the 12L:12D group than those in the 0L:24D group (*P* = 0.02), but there was no significant difference at subjective day between two groups (*P* = 0.22). Additionally, relatively higher levels of IGF-1 over the 28 h period were observed in 12L:12D group (Fig. 6b). Furthermore, the IGF-1 levels were higher in the 12L:12D group than those in the 0L:24D group at both subjective day and night (*P* = 0.00; *P* = 0.04, respectively) (Fig. 6d).

## Discussion

In this study, the effect of 12L:12D of monochromatic green light on hatching time and hatching performance of White Leghorn chickens were determined and the possible underling mechanism involved with the pineal gland was investigated.

In order to evaluate the impact of the monochromatic green light on hatching time, average hatching time and time to 90% hatch were compared. Overall, monochromatic green light stimulation shortened the hatching time. Similarly, Tong et al. [9] reported a 3.4-h shortened hatching time in broilers under monochromatic green LED light stimulation. Decreased hatching time was earlier linked to increase in hatching weight, differences in thyroid hormone levels, organ weight and maturity, post-hatching growth, feeding and general behavior [19, 22]. Additionally, shortening the hatching time may result in energy conservation and reduced cost of hatching. However, reduced hatching time is not always beneficial if the hatching period is wider because this subjects early hatched chicks to longer feed and water deprivation [18, 23, 24]. It has been reported that layer chicks with 48 h feed and water deprivation had a lower body weight and decreased concentrations of serum glucose, total protein, and triglycerides up to 56 d [25]. Furthermore, scattered hatching period may impair day-old chick quality and post-hatch performance [26]. Hatch window and peak hatching period, representing the intervals and concentration of hatching time of a batch of hatched chicks respectively, were not affected by the light stimulation during embryogenesis as observed in the present study. The hatch window was less than 34 h in both the treatment and the control groups. Meanwhile, the external light stimulation did not impair hatchability and chick quality. Taken together, the monochromatic green light shortened the hatching time without impairing hatching performance.

Unlike in mammals, avian pineal gland is an important component of the photo-endocrine system containing both photoreceptors and circadian oscillators. The chicken pineal gland during embryogenesis, in contrast to the post-hatching period, was characterized by a follicular structure [27]. Previous histological study demonstrated that the follicle and its lumen markedly varied in size as well as its wall thickness during embryogenesis [28]. Thus far, the pineal histological changes during embryogenesis in chickens are still unclear, especially for embryos incubated with light stimulation. In the present study, we observed a trend that follicle area and wall thickness increased with advancing embryogenesis, while lumen area increased on d 10 to 14 post incubation and decreased thereafter. This is similar with the reports of previous studies that linked larger follicle areas, thicker walls and limited lumens of pineal gland with advancement in embryonic development in chickens [28, 29]. In this study, conspicuous structural differences observed in embryos under light stimulation were found on d 10 and 12 post incubation. In a recent investigation, Petrusewicz-Kosińska et al. [30] reported an increase in follicle size and wall thickness of pineal gland which were asserted to be a product of proliferation of the cells which create inner and outer parts of follicle wall. Taken together, these results suggest that light may improve early pineal development by promoting the proliferation of specific cells in pineal gland and its attendant morphological structure.

Additionally, environmental factors can also accelerate the maturation of pineal rhythmic function mainly by targeting components of intracellular oscillator [31]. The components of intracellular oscillator have demonstrated little variation among various cell types [32]. CLOCK and BMAL1 are circadian clock proteins, which heterodimerize and bind promoter E-box elements to activate transcription of cryptochrome (*CRY*) and period (*PER*) genes. Sufficiently high levels of *CRY* and *PER* lead their nuclear translocation and subsequent interaction with the heterodimer of CLOCK:BMAL1 to inhibit their transcription [33]. Knockdown of *CLOCK* has been shown to cause a decrease in AANAT activity, demonstrating that AANAT is also an essential component of the circadian clock [34]. In the present study, the expression of clock-related genes including *BMAL1*, *CLOCK*, and *CRY1,* as well *AANAT* exhibited significant circadian rhythm under the 12L:12D light stimulation, but not under the constant dark condition. In tandem with the result of the present study, Kommedal et al. [35] reported a clear 24 h rhythm in the expression of *AANAT* and *CLOCK* genes following 12L:12D light stimulation during embryogenesis; Archer et al. [36] linked 12L:12D photoperiod during incubation with a long-lasting effect on the diurnal rhythms of behavior, which may relate to the rhythmic expression of circadian clock components. These results indicate that light stimulation during incubation may accelerate the maturation of pineal function on circadian rhythm.

In this study, monochromatic green light stimulation promoted the secretion of IGF-1, which is mainly secreted by liver. The advantage on liver development was indeed observed in the 12L:12D group in this study. IGF-1 as a growth factor was reportedly involved in both the proliferation and differentiation of myoblasts [37], satellite cells [7] and pancreatic islets [38]. Previous studies reported elevated levels of thyroid hormone, IGF-1, and growth hormones and their association with either embryonic or post-hatch growth in broiler chickens [39, 40]. Lu et al. [41] suggested that IGF-1 may play important roles in accelerating embryo development. The liver, as the main organ for secreting IGF-1, plays a key role in fat storage absorbed from the yolk at the later stages of embryonic development. It was reported that antioxidant status is a vital aspect of chicken embryo liver development [42]. Melatonin, the main product secreted by the pineal gland, is known as an effective antioxidant [43]. Its exogenous application improved antioxidant status of hepatocytes [11] and was also reported to promote the hepatocyte proliferation and IGF-1 secretion *in vitro* [44]. Following the observation of light-induced changes in morphological structure and rhythmic function of pineal gland, we hypothesized that the MT concentration may be perturbed. Accordingly, results showed elevated serum MT concentration in embryos incubated under light stimulation during subjective night. Interestingly, we observed a consistent trend of plasma MT levels with the rhythmic expression of *AANAT* in this study. Both MT concentration and *AANAT* expression were high during subjective night, but relatively lower during subjective day period. This suggests that MT may play a central role in the development of chick embryo by promoting proliferation of hepatocytes (heavier liver) and consequent upsurge in IGF-1 secretion. However, further research is needed to determine whether the increase in MT concentration, its rhythmic secretion, or both as the cause of the elevated serum IGF-1. Nevertheless, pineal gland is suggested to be the key responsive organ in the lighting process that indirectly regulates embryo development through the increase in the synthesis and secretion of IGF-1 in chicks’ liver via secretion of MT.

## Conclusions

Our results here indicate that the 12L:12D monochromatic green light stimulation shortens the hatching time without impairing hatching performance. Early pineal development was promoted by the 12L:12D monochromatic green light stimulation, as evidenced by modulating histological structure (including follicle area, wall thickness and lumen area) and accelerated maturation of rhythmic function. This further promote the secretion of MT with consequent increase in IGF-1 secretion in livers, which promotes subsequent embryonic development and shortening of the hatching time.

## Supplementary Information

**Additional file 1: Table S1**. Primer sequences for qRT-PCR assays of target and reference genes.

**Additional file 2: Fig. S1**. Incubator temperature over d 7 post incubation of two incubators of each group.

## Data Availability

The data supporting the conclusions of this article is included within the article and its additional file.

## Abbreviations

LED: Light emitting diode
IGF-1: Insulin-like growth factor 1
BMAL1: Brain and muscle ARNT-like-1
CLOCK: Circadian locomotor output cycles kaput
CRY1: Cryptochrome-1
AANAT: Arylalkylamine N-acetyltransferase
FL: Follicle
LM: Lumen
WT: Wall thickness
ZT: Zeitgeber time
GAPDH: Glyceraldehyde-3-phosphate dehydrogenase
MT: Melatonin
CT: Circadian time
CRY: Cryptochrome
PER: Period
