# Supplementary material for: Monochromatic green light stimulation during incubation shortened the hatching time via pineal function in White Leghorn eggs
Source: J Anim Sci Biotechnol. 2021 Feb 2;12:17. doi: 10.1186/s40104-020-00539-x (PMC7856796; doi:10.1186/s40104-020-00539-x)
Supplement: Supplementary file 1 — Additional file 1: Table S1. Primer sequences for qRT-PCR assays of target and reference genes. [file 40104_2020_539_MOESM1_ESM.docx]

**Table S1. Primer sequences for qRT-PCR assays of target and reference genes**

| **Genes** | **Accession no.** | **Primer sequences (5ʹ→3ʹ)** | **Product length, bp** |
| --- | --- | --- | --- |
| *BMAL1* | NM_001001463.1 | F: GGAGCAGCTATCTTCTTCTGACAC | 114 |
|  |  | R: GCTCCAGAACATAGTCGTGATGG |  |
| *CLOCK* | NM_204174.2 | F: GCTTCCAGGTAATGCTCGGAAG | 121 |
|  |  | R: CCAGTCCTGTCGAATCTCACTGG |  |
| *CRY1* | NM_204245.1 | F: GTTATTCGTGGACAGCCAGCAG | 110 |
|  |  | R: GCATCTCTCTCCTTCCCAAACGG |  |
| *AANAT* | NM_205158.1 | F:GAGGATGCCGTCAGCGTGTTC | 85 |
|  |  | R:CGGATCTCATCCAGGTGCAGC |  |
| *GAPDH* | NM_204305.1 | F: ATCACAGCCACACAGAAGACG | 121 |
|  |  | R: TGACTTTCCCCACAGCCTTA |  |
