# Supplementary figures and images for: Monochromatic green light stimulation during incubation shortened the hatching time via pineal function in White Leghorn eggs
Source: J Anim Sci Biotechnol. 2021 Feb 2;12:17. doi: 10.1186/s40104-020-00539-x (PMC7856796; doi:10.1186/s40104-020-00539-x)

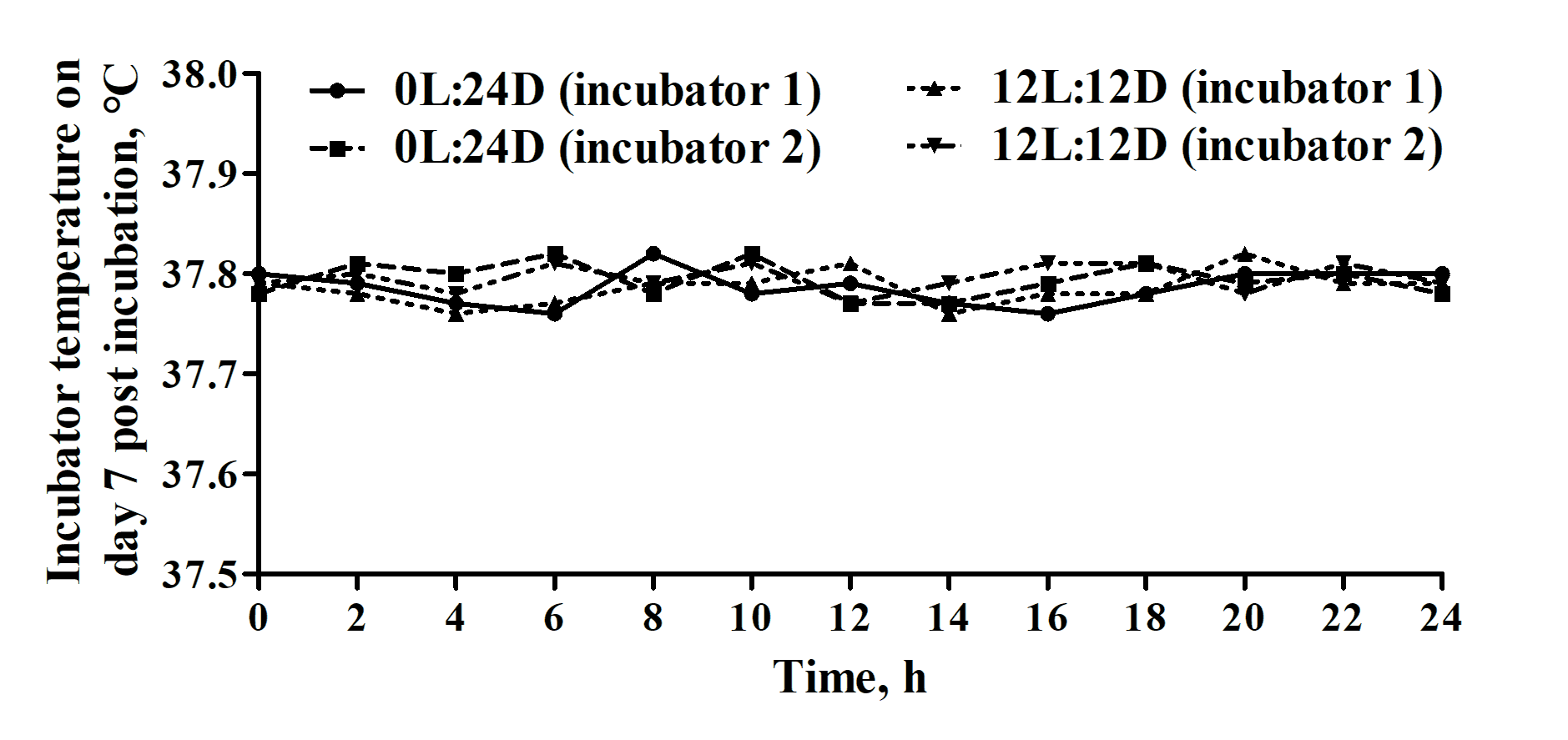

Supplement: Supplementary file 2 — Additional file 2: Fig. S1. Incubator temperature over d 7 post incubation of two incubators of each group. [file 40104_2020_539_MOESM2_ESM.tif]
